# Supplementary material for: Discovery of hierarchical representations for efficient planning
Source: PLoS Comput Biol. 2020 Apr 6;16(4):e1007594. doi: 10.1371/journal.pcbi.1007594 (PMC7162548; doi:10.1371/journal.pcbi.1007594)
Supplement: S1 Appendix — (PDF) [file pcbi.1007594.s001.pdf]

Discovery of Hierarchical Representations for Efficient Planning: S1 Appendix

Momchil S. Tomov[1,2], Samyukta Yagati[3], Agni Kumar[3], Wanqian Yang[4] , Samuel J. Gershman[2]

[1] *Program in Neuroscience, Harvard Medical School, Boston, MA 02115, USA*
[2] *Department of Psychology and Center for Brain Science, Harvard University, Cambridge, MA 02138, USA*
[3] *Department of Electrical Engineering and Computer Science, Massachusetts Institute of Technology, Cambridge, MA 02139, USA*
[4] *School of Engineering and Applied Sciences, Harvard University, Cambridge, MA 02138, USA*

Running title: Hierarchy Discovery for Planning

Corresponding Author:
Momchil S. Tomov
52 Oxford St., Room 295.06
Cambridge, MA 02138
e-mail: mtomov@g.harvard.edu

Supplemental Results

Experiment eight: uncertainty and active learning

With the exception of experiment three, the results so far could also be accommodated by a non-Bayesian hierarchy discovery account that simply searches for the “best” hierarchy as defined by some utility or score function [1]. Indeed, our inference algorithm could be viewed as a form of stochastic search over the space of possible hierarchies, with the (unnormalized) posterior simply serving the role of a score function for comparing candidate hierarchies. Our hierarchical planner also relies on a single point estimate of the hierarchy, which raises the question of whether a

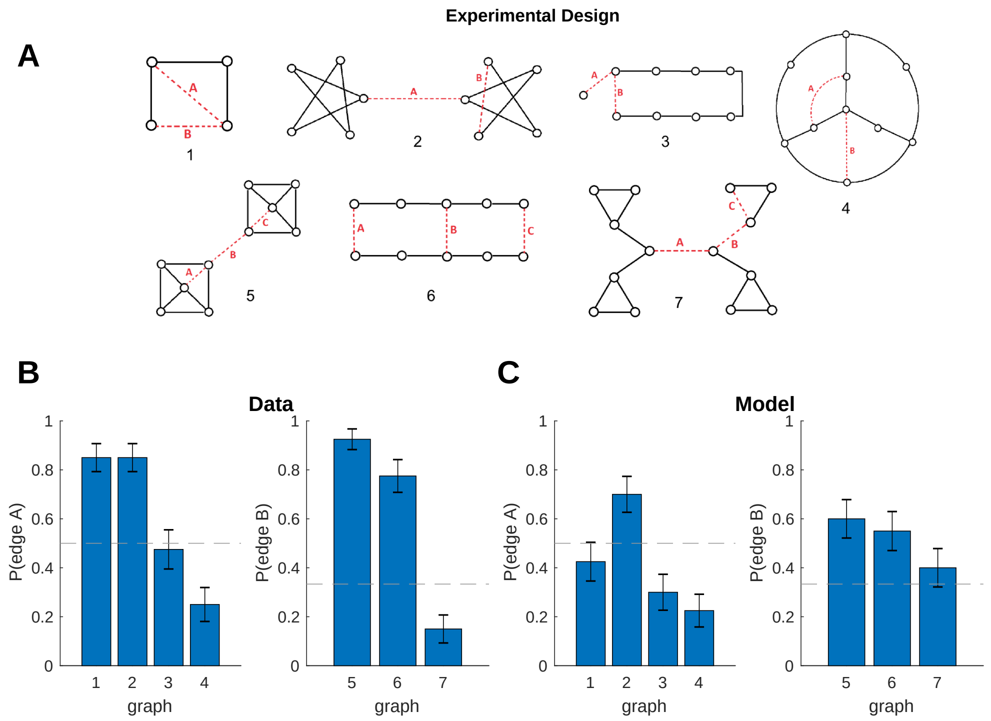

Figure S1. **Uncertainty about hierarchy guides active learning.**

A. The seven graphs shown to participants in experiment eight. Dashed lines indicate edges whose status was unknown. Numbers are graph identifiers.

B. Results from experiment eight showing what fraction of participants chose to learn about edge A in graphs 1-4 (left) and what fraction chose to learn about edge B in graphs 5-7 (right). Dashed line is chance. Error bars are s.e.m. (40 participants).

C. Results from simulations showing that the model exhibited similar preferences. Notation as in B.

Bayesian account is warranted. While the results of experiment three show that the uncertainty in the posterior can affect choices across participants (since the population can be seen as approximating the posterior, with one sample hierarchy per participant), they do not address the question of whether uncertainty is represented at the level of the individual participant.

In this experiment, we sought to explicitly validate the probabilistic aspects of our model by showing that people can make choices based on the uncertainty of the posterior distribution over hierarchies $P(H|G)$. Previous work has demonstrated that both humans and animals have access to their uncertainty [2], which they can report explicitly or leverage to direct their exploratory choices towards informative options [3]. Most related to our study is work on active learning [4, 5] showing that people choose to learn in a way that maximally reduces their uncertainty about the hidden structure of the environment. Applied to our model, this predicts that people would choose to learn about aspects of the graph that maximally reduce their uncertainty over the hierarchy, which we operationalize as the entropy of the posterior (see Methods).

Participants

We recruited 40 participants from the Harvard community. The experiment took around 5 minutes and participants were not paid for their participation.

Design

We presented 40 participants with 7 graphs (“subway networks”; Figure S1A). In each graph, all but two or three of the edges were observable (those edge were described as “tracks possibly under

construction"). We then asked participants to indicate which of the currently unobservable edges they would like to observe (i.e., to learn whether the edge is present or not). Participants could only pick a single edge for each graph.

Procedure

Each participant was given a sheet of paper with instructions and the graphs in Figure S1A. The graphs were presented in a different order. The instructions were as follows:

Imagine that you are a tourist visiting a new city and want to use the subway system to get around. Unfortunately, the subway map you have is out of date, and several tracks on it are marked as "Under Repair". As it might help get you around faster, you decide to ask a passer-by if any of these tracks have finished repair work. Each of the images below represent a subway network, with the circles as stations and lines as tracks. For each subway system, there are 2 or 3 tracks in red, representing tracks that are possibly under construction. Circle ONE track whose repair status you want to find out about the most. There is no need to think too hard about each map!

Results and discussion

If participants only care about the flat graph G, then they should be indifferent between the available choices since they all yield exactly one bit of information. Furthermore, if participants maintain a single point estimate of the hierarchy without keeping track of uncertainty, they would have no basis to estimate how informative each choice is for the purposes of hierarchy discovery. In contrast, we found that participants showed a strong preference in 5 of the 7 graphs (Figure S1B;

$p < 0.02$ for graphs 1,2,4,5,6; binomial tests, Bonferroni corrected for 7 comparisons).

The simulated choices of our model were largely in agreement with the empirical results ($r = 0.76, p = 0.05$, Pearson correlation). This suggests that people explore their environment in a way that facilitates hierarchy discovery, in accordance with our Bayesian account. The main discrepancy between our model predictions and the participants' choices was for graphs 1 and 7. This is likely due to other factors also playing a role in human choices, such as graph geometry or connectivity. In addition, since the model parameters were tuned based on simulations of previous studies that did not assess the effects of uncertainty, it could be that the model weighs various aspects of the graph differently from participants when computing the posterior, which would result in different uncertainty estimates. We leave the fine-tuning of model parameters as the subject of future work. In addition, there are several possible alternative explanations of these results. For example, people could be choosing the line that has the greatest potential to reduce the average shortest path between states. Alternatively, participants might have simply tried to enquire about the connections that would lead to isolated subnetworks.

Supplemental Discussion

Cognitive architectures

The earliest attempts to develop a formal theory of planning date back to the work of Newell and Simon [6, 7] who laid the foundational concepts of planning and problem-solving both in human and in artificial intelligence research. Simon [8] framed problem-solving as an interaction between the participant and the environment, and planning as a search through a state space

that represents the structure of the problem, with operators (or actions) performing transitions between states. In parallel, the seminal work of Miller, Galanter, and Pribram [9] highlighted the hierarchical organization of human action plans, which they linked to people's highly structured representations of the world. Miller, Galanter, and Pribram [9] also proposed the existence of a fast-access, limited-capacity working memory that loads information from a large-capacity "dead storage" system, concepts later formalized as the short-term and long-term memory stores in Newell, Simon, et al. [7]'s production systems. These earlier attempts led to the development of contemporary cognitive architectures such as Soar [10, 11] and ACT-R [12, 13], which aim to capture all aspects of human cognition. Both of these systems can perform hierarchical problem-solving in complex domains based on subgoals, however the subgoals have to be supplied manually. Chunking (referred to as compilation in ACT-R) is implemented by caching or memoizing the solutions to these subgoals [14].

Our approach builds on concepts developed in this tradition. In our model, hierarchical behavior directly arises from the hierarchical representation of the environment. The two memory systems we assume also mirror the short-term/long-term memory stores in these earlier cognitive accounts. Additionally, the form of action chunking we propose as a future addition to the model (see Future directions) is similar in spirit to the chunking mechanisms in Soar and ACT-R. In principle, our hierarchy discovery method could be integrated with these production systems to allow them to decompose the problem space and identify subgoals automatically.

Information-theoretic approaches

Closely related to our study is work by McNamee, Wolpert, and Lengyel [15] proposing an alternative approach to hierarchically decomposing the environment for planning under working memory limitations. Similarly to our proposal, they divide the state space into clusters (or modules) and assume planning first occurs at a high-level (across modules), and is subsequently refined at a lower level (within modules). They define an optimal modularization of the state space as the one which minimizes the expected information-theoretic description length of planning trajectories. Intuitively, this means that the average hierarchical plan in the modularized state space is as simple as possible. Unlike the analysis of Solway et al. [16], this method does not require knowing the optimal behaviors in advance and can also accommodate different task distributions. This implies that it could account for effects based on graph topology and task distribution (see Table 2 in the main text). Unlike our model, it does not account for effects of the reward distribution and uncertainty. By framing the process of hierarchy discovery in terms of Bayesian inference, our model can be used to make predictions about how beliefs evolve during learning (see experiment three and Future directions), and how plans and choices will change correspondingly. Performing Bayesian inference incrementally in this way can also be used to investigate the neural correlates of hierarchy discovery and to understand the underlying neural computations (Figure S2E,F). Indeed, our model does not appeal to a strict definition of optimality (in the sense of producing a hierarchy that is provably optimal), and hence the two approaches can be seen as complementary, with our model explaining how hierarchy is discovered and the analysis of McNamee, Wolpert, and Lengyel [15] validating the hierarchies learned by our model and by people.

Another information-theoretic method for clustering state spaces was proposed by Maisto, Donnarumma, and Pezzulo [17]. Their approach relies on an extension of the CRP that allows clustering based on similarity between states, which can be defined via a prespecified kernel function. Using different kernels, they demonstrate clustering based on bottlenecks, goal states, paths, or other aspects of the graph structure. Their approach relies on algorithmic probability theory to define the kernels (see also [18]). This involves precomputing all possible paths between each pair of states, which renders planning unnecessary. Nonetheless, this approach could provide a useful tool to analyze the optimality of the hierarchies inferred by our model.

Structure learning and other notions of hierarchy

Frank and Badre [19] proposed an alternative notion of hierarchy in terms of action rules at different levels of abstraction [20, 21]. In their framework, low-level rules map stimuli to responses, whereas high-level rules dictate which stimulus dimensions are relevant for the low-level rules. For example, a low-level rule might say “if the traffic light is red, don’t walk”, while a high-level rule might say “when crossing the street, pay attention to the color of the traffic light”. This implements a form of *state aggregation*, which in RL refers to the grouping together of different states and then treating them as a single state, for example by assigning the same values and actions to all states in the group [22, 23]. Note that this is different from state clustering in our model, which still treats each state within the cluster as distinguishable from the rest. By determining which stimulus dimensions are relevant for responding, the high-level rules implicitly render all states with the same value for the particular stimulus dimension as indistinguishable for the purposes of

responding according to low-level rules (for example, “a red light on the sunny day” versus “a red light on a rainy day” both elicit the same response). This drastically reduces the total number of stimulus-response mappings (low-level rules) that need to be learned and allows generalization to previously unseen stimuli.

The connectionist model proposed by Frank and Badre [19] implements error-driven learning at these different levels of abstraction in parallel loops that map onto the corticostriatal hierarchy, with more anterior regions representing rules at increasing levels of abstraction. Despite its ability to account for a range of behavioral and neural data, this approach is fundamentally restricted to learning stimulus-response mappings only, and as such falls into the category of model-free RL since it has no notion of a transition structure over which to plan. As discussed previously, model-free RL – even with state aggregation – cannot account for the results of experiments one through five since the predominant response ($6 \rightarrow 5$) was never reinforced, nor would it support the kind of goal-directed planning presented here. In fact, state aggregation would likely not be possible in these experiments, since there is only a single stimulus dimension (the name of the current station). In their model, the term hierarchy is used to denote a hierarchy of rules that amounts to a compressed mapping from one stimulus to one action. This is fundamentally different from our notion of a hierarchy, in which a hierarchy of states supports the flexible generation of multistep action plans that achieve distant goals. It also differs from the traditional notion of HRL, which refers to a temporal hierarchy, with options consisting of sequences of primitive actions. While in theory the notion of a high-level rule could be extended to include options, the model they propose can only learn to ignore stimulus dimensions and as such can only

compress stimulus-response mappings, whereas to the contrary, options require an expansion of the stimulus-response space.

Partial observability

Closely related to state discovery models is work in RL on partially observable environments [24]. In this scenario, the agent never directly observes its current state but must instead infer it from observations as it interacts with the environment. Formally, the environment is represented by a partially observable Markov decision process (POMDP) in which states, actions and rewards are represented similarly to MDPs, with the key difference that states additionally generate observations. The agent then uses these observations to infer a probability distribution over states – the *belief state* – which it uses for decision making. Building on RL and Bayesian principles, POMDPs provide a normative way to maximize reward under uncertainty. Correspondingly, they have been used to account for a wide range of behavioral and neural results in the animal learning and decision making literature [25, 26]. Recently, neurophysiological evidence from rodents [27, 28, 29] has shown that midbrain dopaminergic firing is consistent with a RL signal computed over such a belief state, thus grounding the POMDP framework in the well-established brain circuits for reward-based learning.

Our model can be seen as an extension of the POMDP framework, with clusters (high-level states in H) acting as hidden states and low-level states in G acting as observations. However, our model differs from the standard POMDP definition in two ways. First, as latent cause models, POMDPs assume observations are independent given the state, whereas our model relies on the relations

between states (E) in order to infer the clusters. Second, unlike latent cause models, POMDPs usually assume a prespecified state space, whereas our model allows for a theoretically unbounded number of clusters, recruiting more clusters as dictated by the data. The second property of our model makes it similar to an infinite POMDP [30], which dynamically expands the state space as more observations are acquired. The first way that our model differs from POMDPs suggests that the analogy between observations and low-level states might be inappropriate, and that our model can be better thought of as a particular kind of infinite hierarchical MDP, in which states are fully observable but there is additional hidden structure which is not observable. Viewed in this way, our model does not support partial observability, a limitation which could be remedied by making the low-level states unobservable and having them generate observations, which would drive inferences about the states, which would in turn drive inferences about the clusters. While this would complicate the inference process, it would bring our model more closely in line with the POMDP framework, making it more applicable in a world in which agents only receive partial information about their state in the environment.

Action chunking and motor sequences

Our work is closely related to the notion of hierarchical control and motor sequencing [31, 32, 33], which studies the behavioral effects predicted by hierarchical action plans. Our work speaks directly to that literature by proposing one particular way in which the representations that support such hierarchical planning might be learned. Indeed, our hierarchical planner is reminiscent of the tree traversal process described by Rosenbaum, Kenny, and Derr [31], and our reaction

time analysis suggests that participants indeed executed sequences of actions in accordance with hierarchical motor sequencing, with action plans generated according to the hierarchical representations predicted by our model.

Our work is also intimately related to a broad literature on chunking in sequence learning [34], also referred to as action chunking. Action chunking refers to the “gluing” of consecutive actions that are reinforced repeatedly into a stereotyped action sequence that is executed as a single behavioral unit. One of the most robust findings in the animal learning literature is the emergence of such stereotyped action sequences after extensive training on a particular task. It is thought to occur as control is transferred from a goal-directed system that chooses actions based on their anticipated consequences to a habitual system that executes entire action sequences in response to perceived stimuli [35].

Action chunking has a distinct neural signature, with bursts of neural activity emerging at key choice points as an animal becomes proficient at a particular task [36, 37, 38]. This so-called *task-bracketing* activity first appears in prelimbic cortex – often associated with goal-directed behavior – and then gradually shifts to infralimbic cortex and dorsolateral striatum – often associated with habitual behavior [39]. Task-bracketing has also been measured in midbrain dopaminergic neurons that project to striatum [38], with a difference between the fraction of direct and indirect pathway neurons that code for the initiation and termination of action sequences [40]. Neural activity representing action sequence boundaries has also been measured in striatum and prefrontal cortex of macaques [41, 42]. Similar task-bracketing activity has also been observed in songbirds [43] and humans [44, 45], suggesting a conserved neural mechanism. One interpretation of these

results is that task-bracketing activity reflects start/stop signals that gate overtrained action sequences, particularly since it appears to be causally involved in the initiation and termination of action chunks [46].

Executing entire sequences as single behavioral units could be beneficial if the cost of processing the outcome of each action is outweighted by the benefit of acting fast at the risk of making mistakes [47]. In its current form, our model only captures state chunking (the creation of state clusters), however it can straightforwardly accommodate action chunking using some form of caching or memoization (see Future directions). In fact, our model makes a distinct prediction about the structure of action chunks, namely that they will fall within the boundaries defined by state chunks (Figure S2C in S1 Appendix). In other words, we predict that state abstraction will drive temporal abstraction: agents will first carve up their environment into clusters of states (state chunks), which in turn will constrain the sequences of actions (action chunks) that are learned, which in turn will operate within the state chunks. This stands in contrast to accounts which assume that the agent first learns useful action sequences and then learns state representations consistent with those sequences post-hoc [48]. This translates into specific predictions about the neural activity of brain regions thought to support action chunking.

Neural implementation

We speculate about how the computational processes proposed here might be implemented in neural circuits, and which brain regions might perform the computations. We also simulate within-trial and across-trial neural signals that could be used to identify the key brain areas involved in hierarchy

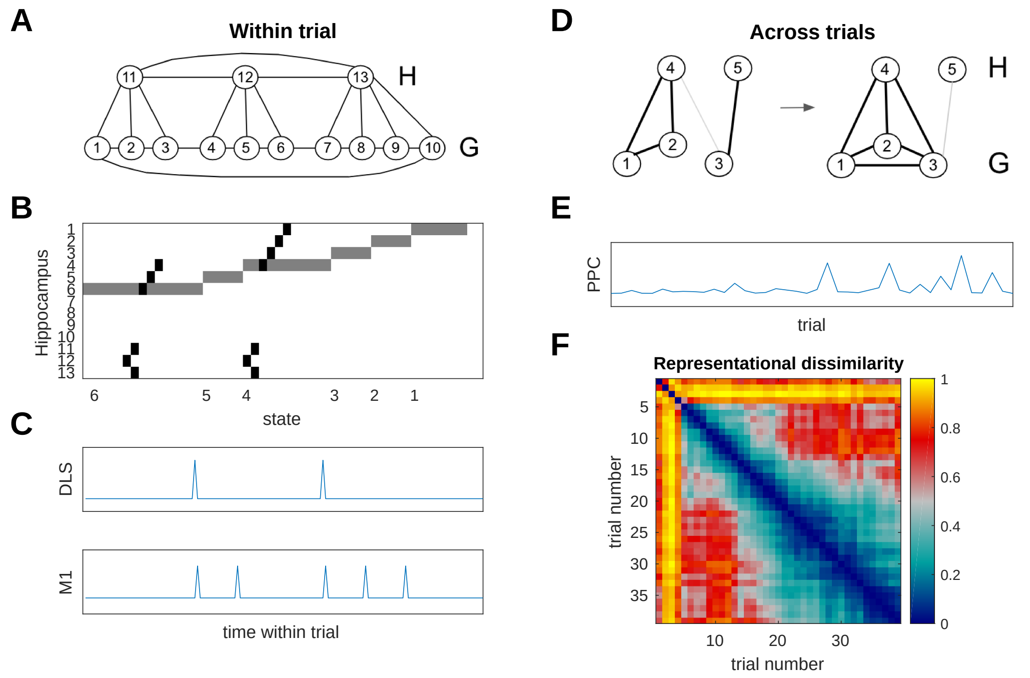

Figure S2. **Hierarchy discovery and hierarchical planning in the brain.**

A. Example neural circuit encoding the low-level graph G (bottom), the high-level graph H (top), and the cluster assignments c from experiment one (Figure 9A). Circles denote units representing graph nodes. Lines denote bidirectional excitatory synapses representing edges and cluster assignments. Number are unit identifiers.

B. Example (idealized) circuit activity during the test trial $6 \rightarrow 1$. Each row represents the activity of the corresponding unit over the course of the trial. States along the X-axis denote the current state following a transition. Gray denotes intermediate levels of activation representing the current state of the agent, akin to hippocampal place cell activity. Black denotes high levels of activation during planning, akin to hippocampal preplay.

C. (Top) Example (idealized) “start” activity at the initiation of each action chunk in dorsolateral striatum (DLS), with action chunks assumed to fall within the boundaries of the state chunks (clusters) in A. (Bottom) For comparison, primary motor cortex (M1) activity at key presses corresponding to transitions. Time course corresponds to B.

D. Example neural circuit illustrating hierarchy discovery via local Hebbian plasticity. (Left) Low-level graph with a single edge (1,2) has nodes 1 and 2 assigned to cluster 4 and node 3 is assigned to cluster 5. (Right) Observing edges (1,3) and (2,3) causes transient activation of nodes 1,2,3 and cluster 4, strengthening the connection between node 3 and cluster 4 and hence reassigning node 3 to cluster 4.

E. Simulated Bayesian update of the (approximate) posterior $P(H|D)$ over the course of learning the graph from simulation four (Figure 7A), which could take place in posterior parietal cortex (PPC).

F. Representational dissimilarity matrix showing the difference in the (approximate) posterior $P(H|D)$ between pairs of trials during the same simulation as in E.

discovery and hierarchical planning.

First, consider the flat graph G only. A straightforward way to encode G in a neural circuit would be to have a single unit (a neuron, such as a place cell, or an ensemble of neurons) represent each node $u \in V$ and excitatory synapses between pairs of units (u, v) represent the edges E (Figure S2A, bottom). The graph structure could be learned via local Hebbian plasticity: when two units are activated right after each other (for example, during a transition between the corresponding states), the synapse between them is potentiated. In order to perform (flat) BFS to find the shortest path from node s to node g, an external input can successively probe each neighbor of s by transiently activating the corresponding unit, triggering a "forward sweep" of activation that propagates through the circuit until it reaches and activates the unit of the goal state g. The neighbor of s that activates g in the least amount of time is the next node along the shortest path to g, so the agent can then physically transition to that state and repeat the process again and again, until finally reaching the goal state g. Assuming some form of short-term synaptic depression or ion channel inactivation that prevents the sweep from going backwards [49], this implements precisely BFS. Similar schemes have been proposed to support forward trajectory planning in the hippocampus [50, 51].

The hierarchical graph H could then be incorporated into the circuit by designating its own set of units and synapses, corresponding to the nodes V' and edges E', respectively. The cluster assignments c could also be implemented as synapses between the units of G and the units of H. In an elegant way, the inferred hierarchy would be isomorphic to the neural circuit that represents it (Figure S2A). This could straightforwardly extend to deeper hierarchies and is consistent with

the presence of place cells and grid cells with different receptive field sizes in the hippocampus and entorhinal cortex [52, 53]. HBFS can be implemented in the exact same way as BFS, with the difference that now the forward sweep can take "shortcuts" through the higher levels of the hierarchy, thus significantly reducing the time it would take to activate the goal unit g. Note that this performs almost the exact same computation as HBFS, with the small difference that transitions "up" the hierarchy (i.e., computing c_u and c_v on line 1 in Algorithm 1) also count as transitions along the path.

The hierarchy could be learned similarly to G, with local Hebbian plasticity strengthening the synapses between units of H (for example, during boundary transitions) as well as the synapses representing the cluster assignments between G and H. To illustrate this, consider the example in Figure S2D (left), with units 1,2,3 representing nodes in G and units 4,5 representing nodes in H. On the left, there is only one edge between nodes 1 and 2 and hence these two nodes are clustered together ($c_1 = c_2 = 4$), separately from node 3 ($c_3 = 5$). However, once the edges (1,3) and (2,3) are observed (Figure S2D, right), this would transiently activate units 1,2,3 together, which (because of nodes 1 and 2) would transiently activate unit 4, leading to potentiation of the synapse between 3 and 4. Assuming some form of local homeostatic plasticity that constrains the total synaptic weight of each unit, this would weaken the synapse between 3 and 5, effectively reassigning 3 to the same cluster as 1 and 2 ($c_1 = c_2 = c_3 = 4$).

Note that this implements a kind of "soft" hierarchy, with the same node potentially being strongly associated with one cluster and weakly associated with other clusters. This could be one way to take into account a probability distribution over hierarchies rather than a single point estimate.

Indeed, allowing all synaptic weights to have continuous values rather than forcing them to be binary can keep track of probability distributions over the edges E and E' as well. In fact, this could also allow the neural circuit to take into account stochastic transitions: transitions that have low probability will simply have the corresponding synapses potentiated less often, resulting in weaker weights. This addresses the limitation of our graph-theoretic approach to only support deterministic transitions, thus extending the framework to support regular MDPs. The continuous range of the synaptic weights would naturally be taken into account by our "neural" HBFS algorithm: the forward sweep will simply be less likely to propagate through weaker synapses. In effect, this will perform a kind of simultaneous, parallel sampling of an entire set of possible trajectories in a way that is drastically more efficient than sampling trajectories one by one (linear versus exponential time). Investigating the theoretical properties of such a mechanism could be the subject of future work.

A neural circuit with the above-mentioned properties could naturally be implemented in the hippocampus and the surrounding cortex. Hippocampus has long been known to encode locations in physical space [54] and has been hypothesized to encode a cognitive map that applies across various non-spatial domains [55]. Recent studies have shown that this is indeed the case, with encoding of non-spatial task-relevant variables such as sound frequency in rodents [56] and even abstract conceptual domains in humans [57]. The units we hypothesize could thus be implemented in the hippocampus-entorhinal circuit, with HBFS taking the form of hippocampal preplay (Figure S2B) which is known to occur at decision points and is predictive of future behavior [58].

While our model only discovers state chunks, action chunking could straightforwardly be incorporated

by caching (or memoizing) the output of BFS and/or HBFS for regularly occurring subgoals (see Future directions). The acquisition of action chunks after extensive training in animals is associated with the emergence of characteristic start/stop signals in basal ganglia circuits [36, 37, 38, 40, 46]. Our model makes the distinct prediction that action chunks and the corresponding start/stop signals will fall within state chunk boundaries (Figure S2C), rather than be dictated purely by the temporal statistics of action sequences. This prediction could be validated empirically by subjecting animals to similar training and test protocols as our participants while measuring neural activity in dorsolateral striatum.

If the probability distribution over hierarchies is implicitly encoded in synaptic weights, as proposed above, then it would be difficult to read it out directly from neural activity. Alternatively, the distribution could be encoded in neural activity patterns, for example using probabilistic population codes or neuronal sampling [59] . This would be consistent with our previous work [60] which found a neural signature of the Bayesian update of the posterior over hidden structures in a frontroparietal network of brain regions, as well as representations of the full posterior in several brain areas. Our model falls within the framework of structure learning and is thus likely to recruit the same underlying neural mechanisms. This prediction can be tested by generating regressors that track Bayesian updates of the posterior $P(H|D)$ during learning (Figure S2E) and using them to identify neurons or brain areas that might implement the actual hierarchy discovery process. In addition, representational similarity analysis [61] could be used to identify areas that maintain the (approximate) posterior $P(H|D)$ (Figure S2F).

Supplemental Methods

Active learning

Drawing on the active learning framework from the causal inference literature [62, 63], we assume the agent will chose to learn about edges of G in a way that provides maximal information about H. Maximizing information about H is equivalent to minimizing uncertainty about H, which can be quantified as the entropy of H:

$$\mathbb{H}(H|D) = -\sum_{H_{disc}} \int P(H|D) \log P(H|D) dH_{cont} \tag{19}$$

Where $H_{disc} = (V', E', c, b)$ are the discrete components of H, $H_{cont} = (p', p, q)$ are the continuous components of H, and D is the data observed so far. We use $\mathbb{H}$ to denote the entropy of a mixed random variable with discrete and continuous components [64].

Computing the entropy in this way is neither computationally feasible nor psychologically plausible. Following previous authors [65], we assume the agent has a subjective probability distribution over possible hierarchies H which can be represented by a set of samples $[H^{(1)}, ..., H^{(M)}]$ with multinomial probabilities $[p^{(1)}, ..., p^{(M)}]$ ($\sum_m p^{(m)} = 1$). If the samples are drawn from the posterior $P(H|D)$, the agent can approximate the entropy as:

$$\mathbb{H}(H|D) \approx -\sum_m p^{(m)} \log p^{(m)} \tag{20}$$

Note that while this is not a proper estimate of the entropy, it can serve as a basis for rational hypothesis testing. In our simulations, we used MCMC to generate the samples from the (approximate) posterior and set the subjective probabilities according to $p^{(m)} \propto P(H^{(m)}|D) \propto P(D|H^{(m)})P(H^{(m)})$.

We use $a_{u,v}$ to denote the action of observing edge (u,v), i.e. finding out whether $(u,v) \in E$. Since there is no way to know in advance what the outcome would be, the agent has to minimize the expected entropy over the two possible outcomes:

$$\begin{aligned}\mathbb{H}(H|D,a_{u,v}) = {} & \mathbb{H}(H|D,(u,v) \in E)Pr[(u,v) \in E|D] \\ & + \mathbb{H}(H|D,(u,v) \notin E)Pr[(u,v) \notin E|D] \qquad (21)\end{aligned}$$

We can compute the probability of each outcome by marginalizing over H and using the sampling approximation:

$$\begin{aligned}Pr[(u,v) \in E|D] &= \sum_{H_{disc}} \int Pr[(u,v) \in E|H]P(H|D)dH_{cont} \qquad (22) \\ &\approx \frac{1}{M}\sum_{m} Pr[(u,v) \in E|H^{(m)}] \qquad (23)\end{aligned}$$

Where $Pr[(u,v) \in E|H]$ is p or pq, according to the generative model. $Pr[(u,v) \notin E|D]$ is approximated analogously.

The agent then chooses the action that minimizes the expected entropy:

$$a = \underset{a}{\operatorname{argmin}} \mathbb{H}(H|D,a) \tag{24}$$

Neural simulations

The example within-trial circuit activations in Figure S2B in S1 Appendix were generated manually, assuming a hierarchy like the one in Figure S2A (responding to the decomposition in Figure 9A). We assumed HBFS is executed at every cluster boundary and that the entire path within the cluster is traversed in a single action sequence that is executed as a single behavioral unit, akin to an action chunk.

To generate the Bayesian update in Figure S2D, we simulated online inference on the graph from the Towers of Hanoi puzzle (Figure 7A), using a particle filter with $M = 100$ particles $[H^{(1)},...,H^{(M)}]$, each initialized from the prior $P(H)$. We started with an empty graph and added edges one by one, with single edge added on each trial. We approximated the posterior $P(H|D)$ with multinomial probabilities $[p^{(1)},...,p^{(M)}]$, where $p^{(m)} \propto P(H^{(m)}|D) \propto P(D|H^{(m)})P(H^{(m)})$ and $\sum_m p^{(m)} = 1$.

Following our previous work [60], we quantified the Bayesian update after each observed edge by computing the Kullback-Liebler divergence between the multinomial approximation to the posterior before and after the update. We additionally performed 10 iterations of MCMC (as described in the inference section) for each particle after each trial in order to rejuvenate the particles [66]. Similar approximations to online Bayesian inference have been used in previous studies [67]. To generate the dissimilarity matrix in Figure S2E, as in our previous work [60],

we computed the cosine distance between the approximate posterior for each pair of trials in the simulation.

References

1. Fernández, Juan A and González, Javier. *Multi-hierarchical representation of large-scale space: Applications to mobile robots*. Vol. 24. Springer Science & Business Media, 2013.
2. Smith, J David, Shields, Wendy E, and Washburn, David A. "The comparative psychology of uncertainty monitoring and metacognition". In: *Behavioral and brain sciences* 26.3 (2003), pp. 317–339.
3. Gershman, Samuel J. "Uncertainty and exploration". In: *bioRxiv* (2018), p. 265504.
4. Bramley, Neil R, Lagnado, David A, and Speekenbrink, Maarten. "Conservative forgetful scholars: How people learn causal structure through sequences of interventions". In: *Journal of Experimental Psychology: Learning, Memory, and Cognition* 41.3 (2015), pp. 708–731.
5. Bramley, Neil R et al. "Formalizing Neurath's ship: Approximate algorithms for online causal learning". In: *Psychological Review* 124.3 (2017), pp. 301–338.
6. Newell, Allen, Shaw, John Calman, and Simon, Herbert A. "Elements of a theory of human problem solving." In: *Psychological review* 65.3 (1958), p. 151.
7. Newell, Allen, Simon, Herbert Alexander, et al. *Human problem solving*. Vol. 104. 9. Prentice-Hall Englewood Cliffs, NJ, 1972.

8. Simon, Herbert A. “Information-processing theory of human problem solving”. In: *Handbook of learning and cognitive processes* 5 (1978), pp. 271–295.

9. Miller, GA, Galanter, E, and Pribram, KH. *Plans and the structure of behavior.* 1960.

10. Newell, Allen. “Unified theories of cognition and the role of Soar”. In: *SOAR: A cognitive architecture in perspective*. Springer, 1992, pp. 25–79.

11. Laird, John E. *The Soar cognitive architecture*. MIT press, 2012.

12. Anderson, JR. *Rules of the Mind*. 1993.

13. Anderson, John R et al. “An integrated theory of the mind.” In: *Psychological review* 111.4 (2004), p. 1036.

14. Laird, John E, Rosenbloom, Paul S, and Newell, Allen. “Chunking in Soar: The anatomy of a general learning mechanism”. In: *Machine learning* 1.1 (1986), pp. 11–46.

15. McNamee, Daniel, Wolpert, Daniel M, and Lengyel, Máté. “Efficient state-space modularization for planning: theory, behavioral and neural signatures”. In: *Advances in Neural Information Processing Systems*. 2016, pp. 4511–4519.

16. Solway, Alec et al. “Optimal behavioral hierarchy”. In: *PLoS computational biology* 10.8 (2014), e1003779.

17. Maisto, Domenico, Donnarumma, Francesco, and Pezzulo, Giovanni. “Nonparametric problem-space clustering: learning efficient codes for cognitive control tasks”. In: *Entropy* 18.2 (2016), p. 61.

18. Donnarumma, Francesco, Maisto, Domenico, and Pezzulo, Giovanni. "Problem solving as probabilistic inference with subgoaling: explaining human successes and pitfalls in the tower of Hanoi". In: *PLoS computational biology* 12.4 (2016), e1004864.

19. Frank, Michael J and Badre, David. "Mechanisms of hierarchical reinforcement learning in corticostriatal circuits 1: computational analysis". In: *Cerebral cortex* 22.3 (2011), pp. 509–526.

20. Collins, Anne Gabrielle Eva and Frank, Michael Joshua. "Cognitive control over learning: Creating, clustering, and generalizing task-set structure". In: *Psychol Rev* 120 (2013), pp. 190–229. DOI: http://dx.doi.org/10.1037/a0030852.

21. Collins, Anne Gabrielle Eva and Frank, Michael Joshua. "Neural signature of hierarchically structured expectations predicts clustering and transfer of rule sets in reinforcement learning". In: *Cognition* 152 (2016), pp. 160–169.

22. Moore, Andrew W. "Variable resolution dynamic programming: Efficiently learning action maps in multivariate real-valued state-spaces". In: *Machine Learning Proceedings 1991*. Elsevier, 1991, pp. 333–337.

23. Singh, Satinder P, Jaakkola, Tommi, and Jordan, Michael I. "Reinforcement learning with soft state aggregation". In: *Advances in neural information processing systems*. 1995, pp. 361–368.

24. Kaelbling, Leslie Pack, Littman, Michael L, and Cassandra, Anthony R. "Planning and acting in partially observable stochastic domains". In: *Artificial intelligence* 101.1-2 (1998), pp. 99–134.

25. Dayan, Peter and Daw, Nathaniel D. "Decision theory, reinforcement learning, and the brain". In: *Cognitive, Affective, & Behavioral Neuroscience* 8.4 (2008), pp. 429–453.

26. Rao, Rajesh PN. "Decision making under uncertainty: a neural model based on partially observable markov decision processes". In: *Frontiers in computational neuroscience* 4 (2010), p. 146.

27. Starkweather, Clara Kwon et al. "Dopamine reward prediction errors reflect hidden-state inference across time". In: *Nature Neuroscience* 20.4 (Apr. 2017), pp. 581–589. ISSN: 1097-6256. URL: http://dx.doi.org/10.1038/nn.4520.

28. Starkweather, Clara Kwon, Gershman, Samuel J., and Uchida, Naoshige. "Medial prefrontal cortex shapes dopamine reward prediction errors under state uncertainty". In: *Submitted for publication* (2018).

29. Babayan, Benedicte M, Uchida, Naoshige, and Gershman, Samuel J. "Belief state representation in the dopamine system". In: *Nature communications* 9.1 (2018), p. 1891.

30. Doshi-Velez, Finale. "The Infinite Partially Observable Markov Decision Process". In: *Advances in Neural Information Processing Systems 22*. Ed. by Y. Bengio et al. Curran Associates, Inc., 2009, pp. 477–485. URL: http://papers.nips.cc/paper/3780-the-infinite-partially-observable-markov-decision-process.pdf.

31. Rosenbaum, David A, Kenny, Sandra B, and Derr, Marcia A. "Hierarchical control of rapid movement sequences." In: *Journal of Experimental Psychology: Human Perception and Performance* 9.1 (1983), p. 86.

32. Rosenbaum, David A, Inhoff, Albrecht W, and Gordon, Andrew M. "Choosing between movement sequences: A hierarchical editor model." In: *Journal of Experimental Psychology: General* 113.3 (1984), p. 372.

33. Koch, Iring and Hoffmann, Joachim. "Patterns, chunks, and hierarchies in serial reaction-time tasks". In: *Psychological research* 63.1 (2000), pp. 22–35.

34. Sakai, Katsuyuki, Kitaguchi, Katsuya, and Hikosaka, Okihide. "Chunking during human visuomotor sequence learning". In: *Experimental brain research* 152.2 (2003), pp. 229–242.

35. Dolan, Ray J. and Dayan, Peter. "Goals and Habits in the Brain". In: *Neuron* 80.2 (2013), pp. 312–325. ISSN: 0896-6273. DOI: 10.1016/j.neuron.2013.09.007. URL: http://dx.doi.org/10.1016/j.neuron.2013.09.007.

36. Graybiel, Ann M. "The basal ganglia and chunking of action repertoires". In: *Neurobiology of learning and memory* 70.1-2 (1998), pp. 119–136.

37. Barnes, Terra et al. "Activity of striatal neurons reflects dynamic encoding and recoding of procedural memories". In: *Nature* 437 (Nov. 2005), pp. 1158–61.

38. Jin, Xin and Costa, Rui M. "Start/stop signals emerge in nigrostriatal circuits during sequence learning". In: *Nature* 466.7305 (July 2010), pp. 457–462. ISSN: 0028-0836. DOI: 10.1038/nature09263. URL: http://dx.doi.org/10.1038/nature09263.

39. Smith, Kyle and Graybiel, Ann. "A Dual Operator View of Habitual Behavior Reflecting Cortical and Striatal Dynamics". In: *Neuron* 79.2 (2013), pp. 361–374. ISSN: 0896-6273. DOI: 10.1016/j.neuron.2013.05.038. URL: http://dx.doi.org/10.1016/j.neuron.2013.05.038.

40. Jin, Xin, Tecuapetla, Fatuel, and Costa, Rui M. "Basal ganglia subcircuits distinctively encode the parsing and concatenation of action sequences". In: *Nature Neuroscience* 17 (Jan. 2014), p. 423. URL: http://dx.doi.org/10.1038/nn.3632.

41. Fujii, Naotaka and Graybiel, Ann M. "Representation of Action Sequence Boundaries by Macaque Prefrontal Cortical Neurons". In: *Science* 301.5637 (2003), pp. 1246–1249. ISSN: 0036-8075. DOI: 10.1126/science.1086872. eprint: http://science.sciencemag.org/content/301/5637/1246.full.pdf. URL: http://science.sciencemag.org/content/301/5637/1246.

42. Desrochers, Theresa, Amemori, Ken-ichi, and Graybiel, Ann. "Habit Learning by Naive Macaques Is Marked by Response Sharpening of Striatal Neurons Representing the Cost and Outcome of Acquired Action Sequences". In: *Neuron* 87.4 (2015), pp. 853–868. ISSN: 0896-6273. DOI: https://doi.org/10.1016/j.neuron.2015.07.019. URL: http://www.sciencedirect.com/science/article/pii/S0896627315006418.

43. Fujimoto, Hisataka, Hasegawa, Taku, and Watanabe, Dai. "Neural Coding of Syntactic Structure in Learned Vocalizations in the Songbird". In: *Journal of Neuroscience* 31.27 (2011), pp. 10023–10033. ISSN: 0270-6474. DOI: 10.1523/JNEUROSCI.1606-11.2011. eprint: http://www.jneurosci.org/content/31/27/10023.full.pdf. URL: http://www.jneurosci.org/content/31/27/10023.

44. Tricomi, Elizabeth, Balleine, Bernard W., and O'Doherty, John P. "A specific role for posterior dorsolateral striatum in human habit learning". In: *European Journal of Neuroscience* 29.11 (2009), pp. 2225–2232. ISSN: 1460-9568. DOI: 10.1111/j.1460-9568.2009.06796.x. URL: http://dx.doi.org/10.1111/j.1460-9568.2009.06796.x.

45. Herrojo Ruiz, María et al. “Encoding of sequence boundaries in the subthalamic nucleus of patients with Parkinson’s disease”. In: *Brain* 137.10 (2014), pp. 2715–2730. DOI: 10.1093/brain/awu191. eprint: /oup/backfile/content_public/journal/brain/137/10/10.1093_brain_awu191/3/awu191.pdf. URL: +%20http://dx.doi.org/10.1093/brain/awu191.

46. Geddes, Claire E, Li, Hao, and Jin, Xin. “Optogenetic Editing Reveals the Hierarchical Organization of Learned Action Sequences”. In: *Cell* 174.1 (2018), pp. 32–43.

47. Dezfouli, Amir and Balleine, Bernard W. “Habits, action sequences and reinforcement learning”. In: *European Journal of Neuroscience* 35.7 (2012), pp. 1036–1051.

48. Konidaris, George. “Constructing abstraction hierarchies using a skill-symbol loop”. In: *IJCAI: proceedings of the conference*. Vol. 2016. NIH Public Access. 2016, p. 1648.

49. Dobrunz, Lynn E, Huang, Emily P, and Stevens, Charles F. “Very short-term plasticity in hippocampal synapses”. In: *Proceedings of the National Academy of Sciences* 94.26 (1997), pp. 14843–14847.

50. Erdem, Uğur M and Hasselmo, Michael. “A goal-directed spatial navigation model using forward trajectory planning based on grid cells”. In: *European Journal of Neuroscience* 35.6 (2012), pp. 916–931.

51. Gönner, Lorenz, Vitay, Julien, and Hamker, Fred H. “Predictive Place-Cell Sequences for Goal-Finding Emerge from Goal Memory and the Cognitive Map: A Computational Model”. In: *Frontiers in computational neuroscience* 11 (2017), p. 84.

52. Jung, Min W, Wiener, Sidney I, and McNaughton, Bruce L. “Comparison of spatial firing characteristics of units in dorsal and ventral hippocampus of the rat”. In: *Journal of Neuroscience* 14.12 (1994), pp. 7347–7356.

53. Kjelstrup, Kirsten Brun et al. “Finite scale of spatial representation in the hippocampus”. In: *Science* 321.5885 (2008), pp. 140–143.

54. O’Keefe, John. “Place units in the hippocampus of the freely moving rat”. In: *Experimental neurology* 51.1 (1976), pp. 78–109.

55. O’Keefe, John and Nadel, Lynn. *The hippocampus as a cognitive map*. Oxford: Clarendon Press, 1978.

56. Aronov, Dmitriy, Nevers, Rhino, and Tank, David W. “Mapping of a non-spatial dimension by the hippocampal–entorhinal circuit”. In: *Nature* 543.7647 (2017), p. 719.

57. Constantinescu, Alexandra O, O’Reilly, Jill X, and Behrens, Timothy EJ. “Organizing conceptual knowledge in humans with a gridlike code”. In: *Science* 352.6292 (2016), pp. 1464–1468.

58. Dragoi, George and Tonegawa, Susumu. “Preplay of future place cell sequences by hippocampal cellular assemblies”. In: *Nature* 469.7330 (2011), p. 397.

59. Pouget, Alexandre et al. “Probabilistic brains: knowns and unknowns”. In: *Nature neuroscience* 16.9 (2013), p. 1170.

60. Tomov, Momchil S, Dorfman, Hayley M, and Gershman, Samuel J. “Neural computations underlying causal structure learning”. In: *Journal of Neuroscience* 38.32 (2018), pp. 7143–7157.

61. Kriegeskorte, Nikolaus, Mur, Marieke, and Bandettini, Peter A. “Representational similarity analysis-connecting the branches of systems neuroscience”. In: *Frontiers in systems neuroscience* 2 (2008), p. 4.

62. Murphy, Kevin P. “Active learning of causal Bayes net structure”. In: (2001).

63. Tong, Simon and Koller, Daphne. “Active learning for structure in Bayesian networks”. In: *International joint conference on artificial intelligence*. Vol. 17. 1. Citeseer. 2001, pp. 863–869.

64. Nair, Chandra, Prabhakar, Balaji, and Shah, Devavrat. “On entropy for mixtures of discrete and continuous variables”. In: *arXiv preprint cs/0607075* (2006).

65. Steyvers, Mark et al. “Inferring causal networks from observations and interventions”. In: *Cognitive science* 27.3 (2003), pp. 453–489.

66. Chopin, Nicolas. “A sequential particle filter method for static models”. In: *Biometrika* 89.3 (2002), pp. 539–552.

67. Abbott, Joshua T and Griffiths, Thomas L. “Exploring the influence of particle filter parameters on order effects in causal learning”. In: *Proceedings of the Annual Meeting of the Cognitive Science Society*. Vol. 33. 33. 2011.
