# Supplementary material for: Discovery of hierarchical representations for efficient planning
Source: PLoS Comput Biol. 2020 Apr 6;16(4):e1007594. doi: 10.1371/journal.pcbi.1007594 (PMC7162548; doi:10.1371/journal.pcbi.1007594)
Supplement: Attachment — Submitted filename: reviewer responses.docx [file pcbi.1007594.s002.docx]

Dear Dr. Pascucci and Dr. Marinazzo,

Thank you for giving us the opportunity to revise our manuscript. We followed the reviewers’ suggestions and included a number of substantial changes to the manuscript. In particular,

- we added a detailed discussion of why the bidirectional associative account would not explain our data,
- we removed the ambiguous experiment from the main text, and
- we shortened the discussion and methods

Below, we respond point-by-point to the reviewers’ comments (original comments in grey).

Kind regards,

Momchil Tomov

Sam Gershman

Reviewer #1: Planning over a large state space is computationally demanding. The computational cost of planning can be reduced with hierarchical representations, and in certain cases, when the right representations are chosen, these computational savings can come without performance substantial costs to performance. However, the question of how people should and do identify the right abstract representations is an open and important problem. Here Tomov and colleagues address this question by developing a generative hierarchical model of low level states and their transition structure, and inverting this generative graph with to do inference over the abstract representations giving rise to the observed low-level state transitions. The model, is able to capture an impressive array of foundational behavioral findings from previous experiments. In addition, the authors test an impressive array of new model predictions with new experiments in human participants, and, by and large, find that the model provides a good qualitative match to the data.

This work is timely, interesting, and undoubtedly very thorough. Given the amount of material, it is probably no surprise that I have a few concerns with the current manuscript, but my impression is that with some changes this paper will make an impressive contribution to an active and important area of computational neuroscience. A complete listing of my concerns is below.

In several places the authors rule out a flat association-based model – however I have the sense that the authors were really rejecting a straw-man model – one where the associations are simply between two consecutive actions or states. As I read the manuscript I had another, slightly more complex associative model in mind that I don’t think is ruled out by the new experiments. In particular, bidirectional associative chains are built based on training sequences and planning is done over low-level states but using association-based distances (eg. less closely associated states are considered to require a larger transition cost). I understand that this idea wouldn’t solve the computational problems associated with planning, but I’m curious whether they can really rule something like this out in the new behavioral experiments that they provide.

We agree with the reviewer that this model would account for experiments one through five and simulation five. However, it would fail to capture the full range of behavioral findings captured by our model. We added the following paragraph to the discussion section:

“One way to implement action selection using the temporal statistics experienced by the agent is to assume that bidirectional associative chains are built based on training sequences and planning is done over low-level states using association-based distances (that is, less closely associated states are considered to require a larger transition cost). This is similar in spirit to the model proposed by Lynn et al. and could replicate the results of experiments one through five. However, the lack of a hierarchical planning component means that it would fail to account for simulations three and four, and the lack of an explicit hierarchy means that simulation two would also pose a challenge. Simulation one controls for the number of transitions, so an associative account would not detect the boundary transitions. It would also fail to capture the reward-based clustering observed in experiment seven.”

How were the model clustering updates made in the learning task? Was the MCMC re-run completely with each new data point? Clearly, in the human experiment, people are adjusting their abstract state clustering online. The authors seem to pitch the model as something that would occur offline, due to heavy computational demand, and I guess in some sense I wondered whether the ability of people to adjust their posterior over state clustering might actually be evidence against this computationally intensive hierarchical discovery procedure, and instead suggest that something cheaper and simpler is done online.

We added the following clarification to the methods section:

“For experiment three, we ran the MCMC sampler before each probe trial, while for the other experiments we ran it before the test trial. We did this for purposes of efficiency, since we only evaluated predictions on the probe/test trials, and since our computational-level analysis is agnostic to the algorithmic details of hierarchy discovery.”

We also added the following to the discussion section, which we hope addresses the reviewer’s concerns:

“Our treatment of hierarchy discovery is restricted to the computational level of analysis (in the

Marrian sense) [27] and assumes the agent can accurately sample from the posterior over hierarchies P(H|D). Scaling up the model beyond the toy experiments considered here would require relaxing this assumption and instead resorting to approximate Bayesian inference. This points to a fruitful avenue for future research, namely probing the algorithmic details of hierarchy discovery. One

possibility is to take our Monte Carlo sampler as a process model of hierarchy inference in the

brain. This is consistent with previous work casting human probabilistic inference as a form of

Monte Carlo sampling [96, 97, 98, 99, 100, 101]. Of particular relevance are accounts of theory

acquisition during development as a form of stochastic search in theory space [102]. In this light,

hierarchy discovery can be understood as stochastic search in the space of hierarchies that occurs

throughout development and aims to distill the complex structure of the world into a compact

representation that can be used for nimble decision-making. This algorithmic account predicts

a bias towards the prior and autocorrelation in the sampled hierarchies that could be assessed

empirically. A noisy approximate inference process could also potentially explain the quantitative discrepancy between our model and the participants in experiment three.

Even with a sampling approximation, inference in large-scale environments would pose a challenge

to computing the (unnormalized) posterior, which requires iterating over all nodes in the low-level

graph. This means that the complexity of a single update of a single cluster assignment is O(N),

which is very inefficient even for an offline process. This computational burden can be alleviated

by noticing that updating the cluster assignment of a given node mainly affects the posterior

through the immediately adjacent nodes. Thus instead of computing the posterior from scratch

for each node update, the agent can simply adjust the posterior based on local changes (in fact, a

Metropolis-Hastings rule like the one we use here can allow the agent to completely ignore the rest

of the posterior and only consider local changes). Such a factorization of the posterior would bring

the computational complexity of a single node update to O(1). Furthermore, the Gibbs sampler we

use here allows updates to be performed in any order, without sacrificing convergence guarantees.

Overall, this suggests an ecologically appealing account of hierarchy discovery, according to which

agents organize their immediate surroundings into clusters using only local update rules, without considering the rest of the world, yet in doing so they form global representations that can facilitate efficient planning between states that they never perceive in a single instant. The only aspect of the posterior which cannot be factorized is the connectivity constraint (see Methods), which could be addressed, for example, by assigning nodes to adjacent clusters only. Such a cheap and efficient online approximation might also explain how people were able to perform hierarchy inference during our relatively short experiments.”

I don’t see the active learning experiment as providing evidence for the proposed model. I’ve got a few concerns with the interpretation of this experiment. The first is with the premise; based on the description of the experiment I don’t understand why participants would report the subway line that has the largest effect on high level clustering. It seems more reasonable that people would choose the line that has the greatest potential to reduce the average shortest path between states -- and this appears relatively consistent with what participants did. I’m also not convinced that the predictions of the model line up all that well with the data, especially considering the large number of alternative metrics that could be used to provide results similar to the subject data. Given the quality of the rest of the experiments, and the length of the paper, I would actually suggest that the authors remove this section from the manuscript… and if they don’t they should provide a more honest interpretation of the experimental results, which in my mind don’t provide clear evidence for the proposed model.

We are inclined to agree, so we have moved the experiment to the supplement and added the following to its results & discussion:

“In addition, there are several possible alternative explanations of these results. For example, people could be choosing the line that has the greatest potential to reduce the average shortest path between states. Alternatively, participants might have simply tried to enquire about the connections that would lead to isolated subnetworks.”

We also removed references to the experiment from the abstract and discussion, and modified the introduction:

“Finally, experiment eight (Supplemental Results) suggests that people explore in a way that maximally reduces uncertainty about the hierarchy, …”

I’m a bit confused about how the cross-cluster edges in G are conditionally independent of b (the bridges) – I would think that the existence of a bridge necessitates a cross-cluster edge, would it not?

We corrected this mistake in the paper. The model takes this into account properly.

In general, I found the generative model to be a plausible abstraction of real world environments. However, one aspect that I feel uncomfortable with is the hard restriction on group membership (each low level state is part of one and only one high level state). I’m curious whether the authors considered a more continuous (or even compositional) grouping strategy and if so, whether the hard restriction makes different predictions than these alternatives.

Soft cluster membership arises naturally if we relax the assumption that subjects are using a single point estimate to plan. We added the following to the discussion:

“Another limitation of our approach is the hard restriction that each low-level node is a member of a single cluster. This restriction could be relaxed by explicitly building in "soft" cluster membership into the generative model, or by allowing agents to entertain multiple possible hierarchies when planning. While we do not exclude the former approach as a possibility, the latter approach arises naturally from our generative model as the posterior can represent graded beliefs in multiple hypotheses. This is consistent with previous work showing how mixtures of deterministic representations can account for graded effects (Goodman 2008).”

We also added the following regarding compositional hierarchies:

“Clustering these modules will naturally give rise to the kind of compositionality characteristic of natural environments.”

I’m unclear on what the lower case c’s are in sampling statement 13 – it would be helpful if they could be defined and that sampling statement unpacked in the text.

We have clarified that those are the cluster assignments.

The authors set the chinese restaurant prior to be 1, favoring a relatively small number of high level clusters. I’m curious why the authors wouldn’t just infer the CRP prior from the data, rather than set it as a parameter. If it were inferred, rather than fixed, would it take a larger value?

We agree that this parameter could be inferred, however this would introduce additional hyperparameters into our already complicated model, which would simply push the problem one level higher. We chose a fixed parameter value for convenience because it seemed to work across experiments and simulations, but other values might work as well, as would an inferred value. Alternatively, it could be fit from the data, but we preferred not to do that since our main purpose was to illustrate how the same model can explain a wide variety of phenomena with relatively little parameter tuning. We emphasized this in the beginning of the results section:

“Our primary focus is on validating the foundational assumptions behind our model without committing to any particular parameterization. We therefore use the same set of generic handpicked parameters across all of our simulations (Table 1) and focus primarily on reliable qualitative effects that are independent of the particular choice of parameters.”

The behavioral result modeled in simulation 5 was unclear from the description. Participants hit the same button no matter what image they see? I’m not sure I understand why RT under such conditions should relate to the number of abstract state boundaries crossed. There were also some typos in figure legend (8b) so maybe this just requires some clarification.

We have clarified the task:

“This was a self-paced serial reaction time task in which participants had to press a different key combination for each node”

And our assumption:

“While this is not a planning task, we assumed that reaction times for cross-cluster transitions would be slower for the same reason as in experiment three: a cross-cluster transition requires planning in the high-level graph, imposing a kind of context switch cost that would slow RT's”

Page 68: “in all stimuli co-occur together” → but don’t they sequentially fixate them?

We assume they could be fixated in any order. Regardless, the main criticism is that these models do not incorporate action selection:

“More importantly, these models make no predictions regarding action selection, and it is not clear how the learned representations can be used for planning.”

Page 78: I don’t understand why a successor representation would not predict results of the experiments. If a successor stores the state-state transitions – shouldn’t it pick up on the over-representation of within cluster transitions during training? There are some directional issues (eg. when training/testing require going in opposite directions) – so maybe that is why the authors think they couldn’t account for them?

We added the following:

“Our results also cannot be explained by the successor representation (Dayan 1993, Momennejad 2017), an intermediate approach along the model-free/model-based continuum that predicts which states a given policy will visit. This will fail to account for experiment three, in which none of the optimal policies visit state 1, and the policies of the random tasks would not favor the transition 6-7 over 6-5.”

It would be useful if the authors could provide more description of the variables and generative structures in figure 3 B,C&D in the figure captions (rather than just in the text).

We added the variable descriptions to the figure legend.

The term “density” is introduced on page 17 but only explained after the sampling statements are provided several pages later.

We have clarified the term:

“Here we use the term graph density to denote the probability that a pair of nodes are connected by an edge.”

Reviewer #2: The authors propose to cast human hierarchical planning as an on-line visiting - essentially, a multi-level breadth-first procedure - on a non-parametric probabilistic model of the environment and its latent causes structured in clusters on the base of topological properties, reward and task distribution.
The learning of model's structure precedes - maybe, it would be more accurate to say that it proceeds in the background like a continuous updating - the planning execution; the authors assume that a Gibbs-like sampling inferential process can exist that intuitively requires more massive computational resources and longer execution time.
Consistency and plausibility of the approach have been pointed out using both a set of simulations taken from the neuroscientific literature and a series of behavioural experiments where collected data have been statistically compared to the computational results.

This reviewer has just one primary concern, one kind suggestion and a couple of other scientific curiosities (maybe doubts).

Concern: the paper is well-written but, definitely, too long. One would try to group the most significant points of the discursive sections in "General Discussion" and to leave introductive and more descriptive parts to the Supplemental Material, although they are appreciable. Presumably, the paper will likely gain in readability and will remain focused on its principal scientific purpose.

Thanks for these suggestions. We have moved experiment 8 to the supplement. We also moved the section on action chunking from the general discussion to the supplemental discussion.

We hope this addresses the reviewer’s concern. We believe the remaining parts – model-free and model-based reinforcement learning, hierarchical reinforcement learning, option discovery – are crucial in contextualizing our work, and in ruling out those models as potential accounts of our data. The future work section provides a roadmap to how the algorithmic details of this model could be elucidated, which could culminate in a psychologically plausible process model of hierarchy discovery. We feel that it’s better to err on the side of having a longer paper, sine people can always skip parts that they don’t want to read.

Suggestion: The idea to rethink abstraction as an inferential process makes sense, and it is valuable the attention paid in making sure that the theoretical proposal still has a physiological plausibility. Nevertheless, it is a non-binding opinion of this reviewer that the choice of parameters'values, as well as their potential analogy with biological/physiological correlates, deservers to be furtherly illustrated. In this sense, some details could integrate the explanation of the adopted framework.

We chose a fixed parameter value in order to illustrate how the same model can explain a wide variety of phenomena with relatively little parameter tuning. We have emphasized this in the beginning of the results section:

“Our primary focus is on validating the foundational assumptions behind our model without committing to any particular parameterization. We therefore use the same set of generic handpicked parameters across all of our simulations (Table 1) and focus primarily on reliable qualitative effects that are independent of the particular choice of parameters.”

Note that we do not claim physiological plausibility for the parameter values. They were simply chosen to get qualitative fits to the data without doing a lot of curve-fitting. Regarding linking the parameters to biological correlates, we prefer not to further lengthen the manuscript with additional speculations (beyond the many we already have in the paper!) given the current state of knowledge in the field, although we think this is a fascinating and important question for future research.

Curiosities/doubts:
1) One criterion conditioning on cluster determination is the topological structure of the environment. The authors posit that observations topologically highly connected need to be in the same cluster. Although this principle is as useful as intuitive, there might be other properties more salient to take into account, mainly if the goal of the hierarchical model is planning. For instance, a node bridging two different communities belongs to any path connecting them, independently to the couple di nodes chosen as start and goal. In this view, the topological saliency of that node is remarkable.
How much is the presented framework configurable in that sense?

This arises naturally in our framework – the middle node will tend to be clustered separately, or with one of the clusters; regardless, it will be frequently chosen as a subgoal state during planning (see simulation three). So while it is not explicitly designated as a special node according to our generative model, behaviorally it will be significant. If we assume a kind of caching of action chunks that lead to particular subgoal states (see supplemental discussion), its saliency will become explicit.

2) Authors have fixed an order to update the posterior conditioned on all other H's component. Does not Gibbs sampling assume exchangeable the posterior to approximate? What does that order entail?

Gibbs sampling can be performed in any order (Bishop 2006) as long as we are able to sample from the appropriate conditional posteriors, which we are. Exchangeability of the posterior is not necessary; all that is necessary is that we can compute all of the conditional distributions to run Gibbs sampling.

3) Are Schapiro and Solway (one and two through five) both indispensable? Do not they show similar points?

These simulations are important for establishing our model as a unifying framework, since they illustrate clustering based on topology only. In contrast, our experiments only promote clusters based on tasks or rewards, but not based on topology.
