# Supplementary material for: Discovery of hierarchical representations for efficient planning
Source: PLoS Comput Biol. 2020 Apr 6;16(4):e1007594. doi: 10.1371/journal.pcbi.1007594 (PMC7162548; doi:10.1371/journal.pcbi.1007594)
Supplement: Attachment — Submitted filename: responses2.docx [file pcbi.1007594.s003.docx]

Dear Dr. Pascucci and Dr. Marinazzo,

Thank you for giving us another opportunity to revise our manuscript. We followed the reviewers’ suggestions and clarified the choice of sampling order. Below, we respond point-by-point to the reviewers’ comments (original comments in grey).

Kind regards,

Momchil Tomov

Reviewer #2: Substantially, the authors have satisfactorily accepted the main proposed suggestion. Specifically, they decided to move the eighth experiment and a section of discussion - the choice of moving the action chunking section seems very appropriate - to the manuscript's supplement.

However, this reviewer would need a clarification concerning the order chosen for the Gibbs sampling. Established that it can be performed in any order, even though the specified order influences the stochastic process, e.g., determining different mixing times and so affecting efficiency, the question is: why did the authors use this particular order? Would have been equal to start the updating procedure by flipping the presence/absence of an edge in E′? Is there any inferential reason?
This reviewer thinks it is worth to spend a brief comment about this issue, for the sake of completeness.

We added the following clarification to the methods:

“Note that while we chose to sample the components of H in an order that roughly follows the generative model, the exact order is actually irrelevant because we sample to convergence and only take the final sample, and hence any arbitrary order would yield similar results.”

A few typos persist in the text; for instance, in Tab. 2 remains Experiment 8 and presumably should be mentioned its displacement in the supporting information text; in the caption of Fig. 8b it is probably "more slowly" in the place of "more slower"; page 79, item 3, "update " is repeated.

Thank you for pointing those out, we corrected the text.
